# Skipping Multiple Exons to Treat DMD—Promises and Challenges

**DOI:** 10.3390/biomedicines6010001

**Published:** 2018-01-02

**Authors:** Tejal Aslesh, Rika Maruyama, Toshifumi Yokota

**Affiliations:** 1 Department of Medical Genetics, Faculty of Medicine and Dentistry, University of Alberta, 8812-112 St. Edmonton, AB T6G 2H7, Canada; aslesh@ualberta.ca (T.A.); yokotama@ualberta.ca (R.M.); 2 The Friends of Garrett Cumming Research and Muscular Dystrophy Canada HM Toupin Neurological Science Research Chair, 8812-112 St. Edmonton, AB T6G 2H7, Canada

**Keywords:** Duchenne/Becker muscular dystrophy (DMD/BMD), antisense oligonucleotides (AOs), multi-exon skipping, phosphorodiamidate morpholino oligomer (PMO, morpholino), eteplirsen, golodirsen, canine X-linked muscular dystrophy (CXMD), golden retriever muscular dystrophy (GRMD), Clustered Regularly Interspaced Short Palindromic Repeat/CRISPR associated protein 9 (CRISPR/Cas9)-mediated genome editing, actin binding domain (ABD)

## Abstract

Duchenne muscular dystrophy (DMD) is a lethal disorder caused by mutations in the *DMD* gene. Antisense-mediated exon-skipping is a promising therapeutic strategy that makes use of synthetic nucleic acids to skip frame-disrupting exon(s) and allows for short but functional protein expression by restoring the reading frame. In 2016, the U.S. Food and Drug Administration (FDA) approved eteplirsen, which skips DMD exon 51 and is applicable to approximately 13% of DMD patients. Multiple exon skipping, which is theoretically applicable to 80–90% of DMD patients in total, have been demonstrated in animal models, including dystrophic mice and dogs, using cocktail antisense oligonucleotides (AOs). Although promising, current drug approval systems pose challenges for the use of a cocktail AO. For example, both exons 6 and 8 need to be skipped to restore the reading frame in dystrophic dogs. Therefore, the cocktail of AOs targeting these exons has a combined therapeutic effect and each AO does not have a therapeutic effect by itself. The current drug approval system is not designed to evaluate such circumstances, which are completely different from cocktail drug approaches in other fields. Significant changes are needed in the drug approval process to promote the cocktail AO approach.

## 1. Introduction 

Muscular dystrophy (MD) is a group of more than 30 different inherited disorders that are characterized by progressive weakness and degeneration of muscle fibers [1]. Duchenne muscular dystrophy (DMD) is one of the most common single-gene disorders in humans, affecting 1 in 3500–5000 newborn males [2]. The symptoms start to emerge around the age of 2–5 years that includes difficulty in walking resulting in patients being wheelchair-bound by the age of 12–13 in most cases [3]. It is an X-chromosome linked recessive disorder arising due to mutations in the *dystrophin* (*DMD*) gene [4]. The *DMD* gene has 79 exons and an approximately 14 kb long transcript, making it the largest known gene in humans [5]. *DMD* encodes dystrophin protein and is expressed in the sarcolemma of the skeletal and cardiac muscle fibers [6]. Dystrophin is a membrane-supporting protein that connects the muscle fiber cytoskeleton to the extracellular matrix (ECM) [7].

The enormous size of the gene is the reason for it being a hotspot for mutations as compared to other genes [8]. When the triplet codon of the mRNA is not preserved, it mostly leads to the loss of dystrophin and subsequent loss of its function [9]. The mutation is known as an out-of-frame mutation when it disrupts the open reading frame (ORF) and prevents dystrophin from being expressed [9]. Sometimes, the ORF is preserved despite the presence of a mutation, which is known as an in-frame mutation, culminating in a truncated yet functional dystrophin leading to a milder form of the disorder, called Becker muscular dystrophy (BMD) [10]. BMD patients exhibit a later onset of the symptoms with slow progression and have a longer lifespan than DMD patients. In some cases, the symptoms are mild such that the diagnosis is made only in the later stages of life [10]. The most commonly used option to ameliorate DMD symptoms is the administration of a high dose of glucocorticoids [11]. Although this strategy prolongs ambulation, there are extensive side effects deteriorating the quality of the patient’s life [11].

Variation in the severity of the disorder opened gateways to various therapeutics in order to ameliorate the severity to a milder phenotype, which is BMD [12]. For example, patients exhibiting large deletions, sometimes encompassing almost half the gene, are associated with milder cases of BMD [13]. Skipping the mutated exon(s) and/or adjacent exon(s) corrects the open reading frame (ORF), thereby leading to subsequent expression of dystrophin in the sarcolemma is thought of as the molecular mechanism underlying the revertant fibers, rare dystrophin-positive fibers in DMD patients and animal models [14,15]. Exon skipping therapy knocks up (rescues) the target protein using antisense oligonucleotides (AOs) by restoring the ORF [16], although this strategy is not applicable to mutations present in the essential dystrophin domains since the exons cannot be skipped without altering the functionality of dystrophin. Exon skipping has been demonstrated to systemically rescue deletion, duplication, splice site and nonsense mutations in animal models [17,18,19,20,21]. The main aim of exon-skipping therapy is to slow down the progression of DMD by interfering with the splicing events thereby converting the severe symptoms to the milder ones as seen in BMD [16]. Thus, it has been an attractive therapeutic approach to treat DMD and many improvements have been made these years [22]. In 2016, the U.S. Food and Drug Administration (FDA) has conditionally approved Sarepta Therapeutics’ AO called eteplirsen as a treatment for DMD, which targets exon 51 and is applicable for approximately 13% of patients [23].

One limitation of exon-skipping strategy, however, is the limited applicability. In addition, the stability and function of each truncated dystrophin protein are unclear [24]. Importantly, multiple exon skipping (or multi-exon skipping) could address both issues. First, multi-exon skipping is potentially applicable to 80–90% of DMD patients in total [24]. Second, multi-exon skipping enables us to choose the truncated dystrophin protein which is more functional [24]. For example, in screening several truncated dystrophins, it was found that the polyproline structure present in hinge 2 region of the rod domain of dystrophin influences the functional capacity. Replacing this region with hinge 3 significantly improved the functional capacity and prevented muscle degeneration [25]. Recently, multi-exon skipping has been demonstrated in mouse and dog models of DMD using cocktail AOs [24]. In addition, a Clustered Regularly Interspaced Short Palindromic Repeat/ CRISPR associated protein 9 (CRISPR/Cas9)-mediated approach has been employed to genetically remove multiple exons in the *DMD* gene [26,27]. Here, the recent development of multi-exon skipping will be discussed in the coming sections. In addition, we will discuss the regulatory challenges associated with the cocktail AO approach. 

## 2. Advancements in Multi-Exon Skipping Therapy

Restoration of the ORF by employing AOs to remove frame-disrupting exons in order to bypass the mutation and produce a truncated dystrophin protein is the ultimate goal of antisense-mediated exon-skipping [28]. Approximately 70% of DMD patients with deletions and 47% with nonsense mutations are estimated to be treated by single exon skipping, rising to 80–97% by multiple exon skipping [20,29]. Additionally, multi-exon skipping offers the prospect of selecting the truncated dystrophin that optimizes the protein function or stability. For example, *DMD* exons 3–9 deletion and exons 45–55 deletion are both known to be associated with a remarkably mild BMD phenotype compared to smaller in-frame deletions in these regions [29,30,31,32,33,34,35,36]. The systemic effects of antisense-mediated multi-exon skipping tested in some of the models include: *mdx52* mice with exon 52 deletion for multi-skipping of exons 45–55 [17,37,38] and canine X-linked muscular dystrophy (CXMD) dogs for multi-skipping of exons 6–9 [39]. Additionally, a Clustered Regularly Interspaced Short Palindromic Repeat/CRISPR associated protein 9 (CRISPR/Cas9)-mediated approach has been demonstrated to remove *Dmd* exons 52–53 systemically in *mdx4cv* mice with a nonsense mutation in dystrophin exon 53 [26]. In the following subsections, we will cover these approaches.

### 2.1. Use of Antisense Oligonucleotides and Phosphorodiamidate Morpholino Oligomers for Single- and Multi-Exon Skipping

**Figure 1:**
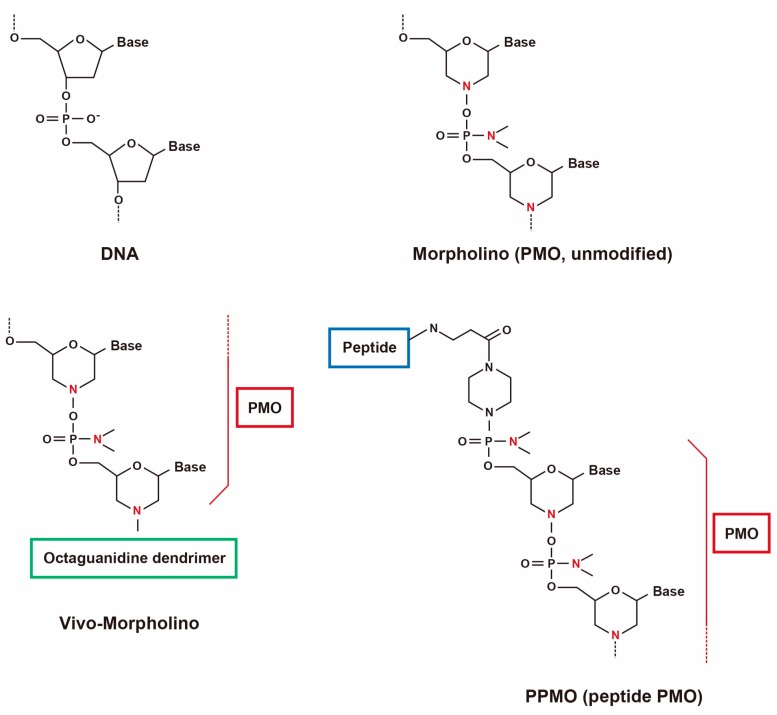
Structures of PMOs and conjugates.

AOs are short, synthetic nucleic acid sequences about 8–50 bp long, that selectively hybridize to target mRNA sequences (Figure 1) [40]. Modifications are made to the phosphate backbone and the sugar rings that can change the solubility, potency, binding and stability (Figure 1) [41]. These modifications increase the affinity of AOs to the target RNA and also protect AOs from nuclease degradation, preventing the interaction of spliceosome machinery with regions of the AO and thereby resulting in the initiation of splicing [42].

Phosphorodiamidate morpholino oligomers (PMOs, morpholinos) are one of the most promising AOs in which chemical modifications are made to the backbone by replacing the phosphodiester backbone by phosphorodiamidate linkages making PMOs unrecognizable to the nucleases thus greatly enhancing the stability [43]. Previous experiments carried out using PMOs have successfully demonstrated the restoration of dystrophin expression in multiple muscle groups following systemic delivery in several murine and dog models [44,45,46].

Use of AO therapy in DMD has an advantage compared to its use in other diseases as dystrophic fibers take up more AO compared to the healthy fibers [47]. In addition, recent studies show that the efficiency of PMO delivery into the muscle depends firstly on the accumulation and retention of PMO within the inflammatory foci associated with dystrophic lesions and secondly on the fusion of myoblasts containing PMO into the repairing myofibers [48]. 

Eteplirsen or Exondys 51 (Sarepta Therapeutics, Cambridge, MA, USA) is a PMO that selectively binds to exon 51 of the pre-mRNA and restores the open reading frame by inducing exon-skipping and produces a truncated protein [49]. It was conditionally approved by the Food and Drug Administration (FDA) in 2016 [23]. Eteplirsen rescued dystrophin levels in the range of 0.28% of healthy muscle, in contrast to the expected value of 10% for regaining muscle function [23]. Three years after the administration of eteplirsen at 30 mg/kg and 50 mg/kg, no adverse effects, immune activation or hypersensitivity have been reported [23]. However, the FDA’s approval of eteplirsen remains controversial because the approval has a weak evidence supporting its effectiveness in terms of restoring dystrophin expression [23]. Several PMOs targeting other *DMD* exons, including golodirsen (SRP-4053, Sarepta Therapeutics, Cambridge, MA, USA) and NS-065/NCNP-01 (NS Pharma, Paramus, NJ, USA), are currently under clinical trials that target exons 42, 52, 53 and 55 [50]. By skipping these exons, approximately 28% of DMD patient mutations would be potentially treatable [51].

An advantage of PMOs is that they have a charge-neutral backbone and as such cell-penetrating moiety can be easily conjugated, which is a powerful tool to induce multiple exon skipping (Figure 1) [52,53]. Octa guanidine-conjugated PMOs are also called vivo-morpholinos (vPMOs) that possess a cell-penetrating octa-guanidinium dendrimer. vPMOs have shown very efficient splicing modulation in targeting the skipping of *DMD* exons 6 and 8 in dystrophic dogs and exons 45–55 in *mdx52* mice [37,54]. Peptide-conjugated PMOs (PPMOs) have also been shown to efficiently rescue cardiac and skeletal muscles in *mdx* mice and dog models [55,56,57,58,59].

### 2.2. Exons 6–9 Multi-Exon Skipping Using PMOs in the Canine Model

**Figure 2:**
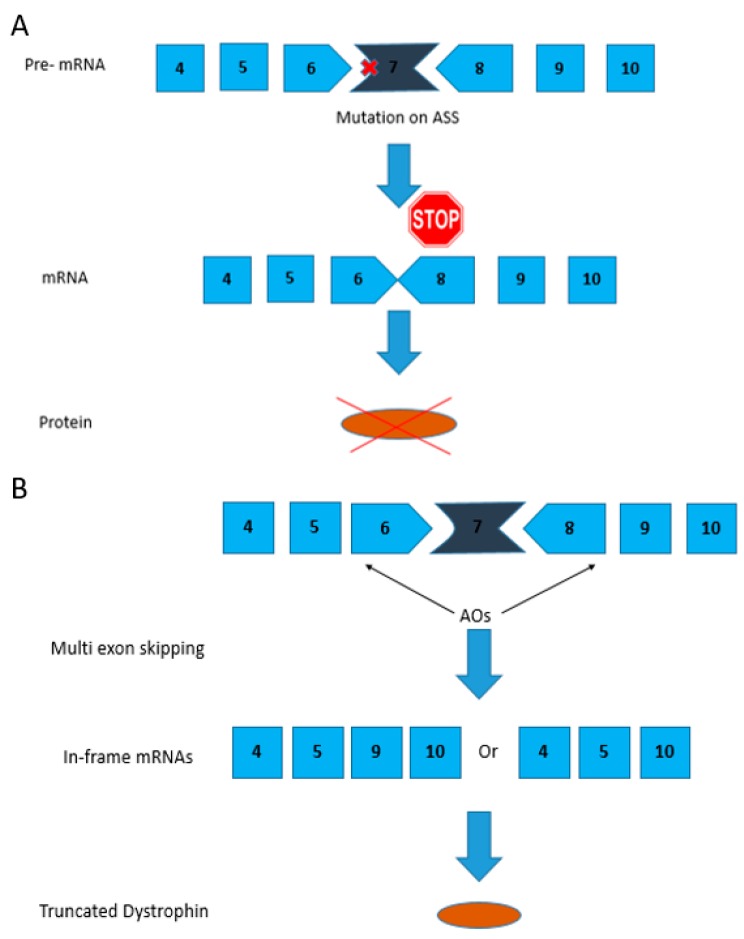
Mutation pattern of the CXMD/golden retriever muscular dystrophy (GRMD) dog models and exons 6–8 skipping strategy using AOs. (**A**) A point mutation in the acceptor splice site (ASS) in intron 6 in CXMD dogs leads to exon 7 being skipped from the dystrophic dog mRNA; (**B**) AOs are designed such that they bind to exons 6 and 8 causing them to be spliced out thereby correcting the reading frame. Exon 9 is spliced spontaneously along with exons 6 and 8.

The first systemic multi-exon skipping was demonstrated in a DMD dog model [60]. Being large in size, dystrophic dogs are more suitable for clinical grading and detailed analysis when compared to other animal models [60]. Dog models have an advantage over mouse models because they represent the human disease more closely [60]. The canine X-linked muscular dystrophy (CXMD) model harbors a point mutation in intron 6 that leads to exon 7 being deleted (Figure 2). Restoration of the ORF requires exons 6–8 being spliced out and hence this model is used to test the efficacy and safety of multi-exon skipping [61].

In this particular study, AOs are designed in such a way that they bind to exons 6 and 8 resulting in them being skipped in order to correct the reading frame as shown in Figure 2. Exon 9 encodes a hinge domain that results in it being spontaneously skipped [45]. A cocktail of AOs (Ex6A, Ex6B and Ex8A) was administered at various dosages to the CXMD dog model. Intravenous injections of the morpholino cocktail of Ex6A, Ex6B and Ex8A with a dosage of 120 mg/kg weekly for 5 weeks showed an increase in dystrophin-positive fibers [39]. Similarly, when the dosage was increased to 240 mg/kg weekly for 7 weeks, an improvement in histopathology was observed. Clinical grading of the morpholino treated dogs also showed an improvement in the walking and running abilities [39]. 

### 2.3. Efficacy of Exons 6–9 Multi-Exon Skipping Using Peptide-Conjugated Morpholinos in the Heart of a Dog Model

PMOs conjugated with peptides (PPMOs) have the ability to penetrate the cell in order to induce dystrophin expression more effectively [58]. In order to test the efficiency and safety of systemic delivery of PPMOs, 3-PPMO with a total concentration of 12 mg/kg was administered 4 times consecutively to CXMD dogs [55]. Using Western blot to compare the dystrophin levels, it was observed that the dystrophin levels in treated dogs increased and equaled to 5% of the total dystrophin levels in WT dogs in cardiac muscles in addition to skeletal muscles [55]. Exons 6–8 were skipped along with the spontaneous deletion of exon 9 which does not disturb the ORF [55]. Also, immunohistochemistry of the myocardium muscles revealed that dystrophin-positive fibers were observed 2 weeks after the last systemic injection [55]. DMD patients show vacuole degeneration in cardiac Purkinje fibers which were supposedly due to the loss of dystrophin. Immunohistochemistry of the fibers revealed amelioration of vacuole degeneration in CXMD dogs where the degeneration was significantly reduced through intra-coronary injections (i.c.) and through intravenous (i.v.) injections [55]. Peptides have the tendency to behave as antigens and can trigger an immune response. However, systemic administration of 3-PPMO did not activate the immune system which was verified by the leucocyte count in treated dogs [55]. Though limited dogs were used for studying the efficacy of intravenous administration of 3-PPMO, no toxicity was reported [55]. Thus, it can be inferred that multi-exon skipping using PPMOs can efficiently restore dystrophin expression without any or minimal immune response [55]. 

### 2.4. Multi-Exon Skipping of Exons 3–9—A Potential Target for Therapy

Exons 3–9 and exons 45–55 are found to be mutational hotspots in the *DMD* gene, covering approximately 7% and 47% of patients, respectively [30,36]. According to a medical case study in 2016, a 27-year-old male exhibited an asymptomatic phenotype with a deletion of exons 3–9 [30]. In spite of having high levels of serum creatine kinase, he did not suffer from muscle atrophy, weakness or developmental delay at the age of 12. At the age of 15, there were minor changes in the size of the muscle fibers, yet there were no signs of necrotic tissues or cellular infiltration. When the patient turned 27, physical examination revealed normal serum creatine kinase levels. It has been proposed that presence or absence of active binding sites (ABS) may be responsible for determining the mildness of the phenotype. There are 3 ABS in the N-terminal domain (ABS 1--3). The patient whose exons 3–9 were skipped lacks ABS 2 and 3 but shows the presence of ABS1. Reports suggest that ABS1 is essential for actin binding ability of dystrophin and necessary for maintaining dystrophin function in the skeletal muscles. Lack of ABS2 and ABS3 can, therefore, be associated with a Becker phenotype. According to a previous study, when there is a mutation in the 5′ region of the gene, around 30–40% of dystrophin levels are essential to prevent dystrophy of the muscles. However, the patient in the current study showed only 15% of the normal levels and yet expressed a very mild phenotype. Contradictions in these findings are a result of different mutational regions. Mutations in the 5′ actin binding region are normally associated with a severe BMD phenotype. From the above case study, it can be concluded that the deletion of exons 3–9 may produce low quantities of the structurally stable protein and that 15% of the normal dystrophin levels are sufficient to maintain muscle integrity given that the protein is functional. Since skipping of exons 3–9 covers the mutational hotspot, it seems a promising therapeutic target. However, further studies are required to test the therapeutic efficiency of skipping exons 3–9.

### 2.5. Functional Correction of Dystrophin Actin-Binding Domain with DMD Exons 3–9 Deletion Using CRISPR/Cas9

Another emerging approach to induce multiple exon skipping is genome editing such as the Clustered Regularly Interspaced Short Palindromic Repeats (CRISPR) system. CRISPR works as an adaptive immune system against phage infection in bacteria by making use of a single guide RNA (sgRNA) that guides the endonuclease to specific genomic sequences resulting in their cleavage [62]. The CRISPR/Cas system is a promising approach towards the correction of many genetic defects [63].

It is reported that patients with exons 3–9 skipped do not display an apparent phenotype making multi-exon skipping of exons 3–9 an effective approach to treat ABD-1 mutations [30]. CRISPR/Cas9 system was used to induce skipping of exons 3–9 in the *DMD* gene in healthy human induced pluripotent stem cells (iPSCs) to analyze the effect of ABD-1 deletions [27]. This was achieved by using and comparing the following 3 strategies:

1. Generating del. Ex3–9 iPSCs by targeting introns 2 and 9 and the subsequent deletion of exons 3–7.
2. Generating del. Ex6–9 iPSCs by targeting introns 5 and 7 and the subsequent deletion of exons 6–9.
3. Generating del. Ex7–11 iPSCs by targeting introns 6 and 11 and the subsequent deletion of exons 7–11.

CRISPR/Cas9 mediated editing strategies produced different modifications where del. Ex3–9 retained ABS1; corrected del. Ex6–9 retained both ABS1 and ABS2; corrected del. Ex7–11 retained all three. However, it is interesting to note that although the open reading frame was maintained by deletion of exons 7–11, it produced the least stable protein and minimal restoration of function due to deletion of amino acids 178–444 that led to protein misfolding and subsequent degradation. The del. Ex6–9 strategy could not fully restore the function to the control levels [27]. The del. Ex3–9 was the most effective of the 3 strategies by generating a truncated protein lacking amino acids 32–320 and restoring the functionality in iPSC-derived cardiomyocytes. In conclusion, this strategy of deleting exons 3–9 can be an ideal candidate targeting 7% of DMD population caused by mutations in ABD-1.

### 2.6. vPMO-Mediated Multi-Skipping of Exons 45–55 in Mdx52 Mice

Most of the patients with an in-frame mutation where exons 45–55 are skipped express a very mild BMD or asymptomatic phenotype [31,32,64]. Interestingly, exons 45–55 of the *DMD* gene cover the mutation hotspot [36]. Therefore, multi-exon skipping of exons 45–55 using AOs is a promising strategy that could treat almost 47% of DMD patients [36]. *Mdx52* mice harbor a deletion mutation of exon 52 and, therefore, is a good model to test exons 45–55 skipping [65]. The efficiency of intra-muscular and systemic administration of vPMOs to skip the entire region comprising of exons 45–55 was tested in *mdx52* mice [37,38]. In order to do so, a dosage of 10-vPMO cocktail targeting exons 45–51 and exons 53–55 with 6–12 mg/kg in total was administered every 2 weeks that showed to efficiently induce skipping of exons 45–55. Histopathology of the muscle revealed amelioration of the muscle and lesser degeneration of muscle fibers. Systemic delivery also ensured improved muscle function with no detectable immunoreaction when compared to non-treated mice. There was no evidence of toxicity after this administration regimen. However, further studies are needed to test dose escalation and reduction as well as chronic toxicity assessments. 

### 2.7. CRISPR/Cas9 for Multi-Exon Skipping Targeting DMD Exons 52–53

**Figure 3:**
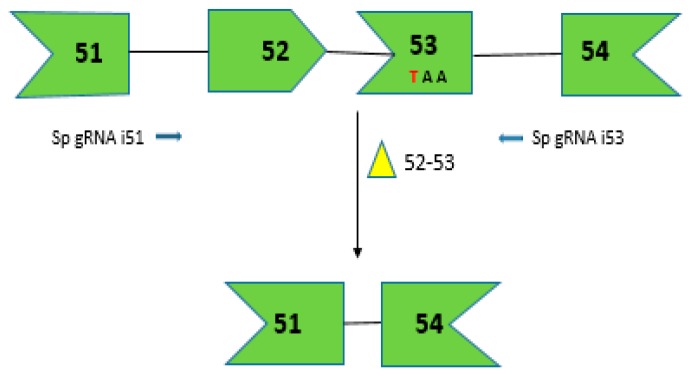
CRISPR/Cas9-mediated gene editing for creating dystrophin mRNA with a corrected ORF by removing the premature stop codon due to C to T transition shown in red. The dual vector approach targets introns 51 and 53 to direct the excision of exons 52 and 53. The arrows depict the sgRNA target sites in the intronic region shown in 5′-3′ direction based on target strand.

In order to assess the efficiency of CRISPR/Cas9 system to remove multiple *DMD* exons, adeno-associated virus (AAV-6)-mediated delivery of CRISPR/Cas9 was used in *mdx4cv* mice [26]. *Mdx4cv* mice harbor a nonsense mutation in exon 53 and at least 2 exons, exons 52–53, need to be removed, making it an appropriate model to test multiple exon skipping (Figure 3) [66]. Due to the limited carrying capacity of AAV (~5 kb), a dual-vector strategy which was to be administered locally into the tibialis anterior (TA) of the mouse model *mdx4cv* [26]. This involved a nuclease vector expressing SpCas9 and a set of vectors consisting of two single-guide RNA (sgRNA) as shown in Figure 3. These sgRNAs direct Cas9-mediated cleavage of DNA within the intronic regions before exons 52 and after exon 53. DNA repair using non-homologous end joining (NHEJ) resulted in the deletion of about 45 kb of genomic DNA and 330 bp in the encoded mRNA [26]. Immunostaining of muscle cross-sections revealed that 41% of the myofibers expressed dystrophin when exons 52–53 were excised using the local delivery dual vector approach which improved the muscle function in the TA. Systemic delivery of the dual vectors ensured dystrophin expression in 34% of the total cardiac myofibers in the heart after 4 weeks post-transduction [26].

Using the dual-vector strategy can be advantageous because of the flexibility by offering variations in the ratio of between the targeting components and the nuclease vector which may enhance efficiency. In vivo transduction of satellite cells (muscle stem cells) can ensure permanent correction of dystrophin protein and thus its continual expression. However, studies using CRISPR/Cas9 system targeting satellite cells are yet to be carried out. Further studies are necessary in order to target mutation hotspots for DMD (e.g., exons 45–55 and exons 3–9) and to ensure the body-wide dystrophin expression. Potential off-targets need to be carefully assessed before every gene-editing strategy to ensure the long-term safety and efficacy [26].

## 3. Future Implications and Clinical Hurdles

Antisense-mediated exon skipping as a therapeutic strategy developed from the mid-90s holds promising therapeutic potential [67]. However, in today’s date, certain barriers are still prevalent especially the nature of personalized therapy, as the mutations causing DMD vary and are unique in DMD patients [29]. Eteplirsen, a drug approved by the FDA, is still holding a controversy regarding its approval whereas another drug drisapersen was rejected. Multiple exon skipping is an attractive approach to overcome the applicability issue. However, the cocktail AO approach faces an additional regulatory challenge [23]. The current regulations require each of the AO cocktail components and all possible cocktail combinations to undergo toxicological testing, creating regulatory barriers that are enormously expensive and intimidating [68]. One must note that not every part of the cocktail is beneficial to every patient (for example, an ideal 11-exon AO cocktail when administered to a patient with a deletion of ≥1 exons, not every part of the cocktail will serve molecular benefits to individual patients) thereby making it an uncharted territory for the FDA [51]. In another example, exons 6 and 8 need to be skipped together to restore the reading frame when DMD exon 7 is deleted (e.g., in dystrophic dogs). Hence, the AOs targeting these exons in combination have a therapeutic effect and individual oligos cannot have a clinical effect by itself. The current drug approval system is not designed to evaluate circumstances like this scenario and requires significant changes to promote the cocktail approach. 

Since AO based exon-skipping therapy targets the pre-mRNA with limited persistence, repeated injections are necessary. CRISPR/Cas9 system, a robust tool for editing DNA, has shown promising results by permanently correcting mutations in the *DMD* gene in mouse models and human-derived iPSCs [26]. However, one of the potential issues with the use of CRISPR/Cas9 system includes the occurrence of off-target cleavage [69]. Although no such concerns were reported by the study that used iPSCs modified by this gene-editing system, AAV-mediated systemic delivery leads to a robust expression for more than 1 year, which will increase the risk [70]. One should also note that Cas9 may pose a safety concern for clinical applications. Nevertheless, CRISPR/Cas9 has shown promising results by correcting mutations in the *DMD* gene. 

While the current clinical trials are focusing on single-exon skipping, the success of multi-exon skipping relies on future investigations and a change in the stance by the regulatory bodies. With continuous developments and modifications in AO-based multi-exon skipping therapy, it can be a promising and safe therapeutic strategy to treat the majority of DMD patients in the future.
